# Multi-directional optical coherence tomography for retinal imaging

**DOI:** 10.1364/BOE.8.005560

**Published:** 2017-11-13

**Authors:** Andreas Wartak, Marco Augustin, Richard Haindl, Florian Beer, Matthias Salas, Marie Laslandes, Bernhard Baumann, Michael Pircher, Christoph K. Hitzenberger

**Affiliations:** 1 Center for Medical Physics and Biomedical Engineering, Medical University of Vienna, Währinger Gürtel 18-20 / 4L, 1090 Vienna, Austria; 2 Institute of Applied Physics, Vienna University of Technology, Wiedner Hauptstraße 8-10/134, 1040 Vienna, Austria

**Keywords:** (170.0170) Medical optics and biotechnology, (110.4500) Optical coherence tomography, (170.4470) Ophthalmology

## Abstract

We introduce multi-directional optical coherence tomography (OCT), a technique for investigation of the scattering properties of directionally reflective tissue samples. By combining the concepts of multi-channel and directional OCT, this approach enables simultaneous acquisition of multiple reflectivity depth-scans probing a mutual sample location from differing angular orientations. The application of multi-directional OCT in retinal imaging allows for in-depth investigations on the directional reflectivity of the retinal nerve fiber layer, Henle’s fiber layer and the photoreceptor layer. Major ophthalmic diseases (such as glaucoma or age-related macular degeneration) have been reported to alter the directional reflectivity properties of these retinal layers. Hence, the concept of multi-directional OCT might help to gain improved understanding of pathology development and progression. As a first step, we demonstrate the capabilities of multi-directional OCT in the eyes of healthy human volunteers.

## 1. Introduction

Conventional optical coherence tomography (OCT) employs a single illumination/detection channel to obtain one-dimensional depth profiles (A-scans) of a sample along the direction of the incident beam. However, to satisfy a variety of demands for different OCT applications, additional measurement channels have been introduced. Multi-channel OCT was applied, amongst others, for the aim of speckle reduction [1–4], image range extension using multiple foci [5, 6] as well as full range detection [7, 8], imaging speed enhancement (parallelization) [9–12], vibrometry [13] and functional OCT – in particular for OCT angiography (OCTA) [14–16] and Doppler OCT (DOCT) [17–21].

**Fig. 1:**
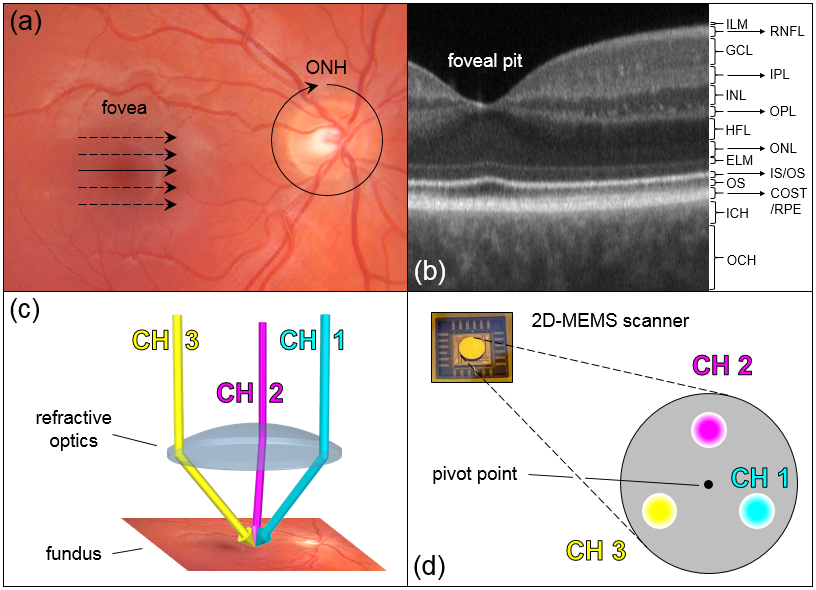
(a) Fundus photo of a healthy eye indicating the two retinal regions of interest (ROIs) investigated in this work: the perpapillary (including the ONH) and the macular region (including the fovea). The applied scanning patterns per ROI are indicated by black arrows (ONH: circumpapillary (CP); fovea: linear and raster). (b) Representative linear scan through the macular region of the same eye. Retinal and choroidal layers (labelled according to [36]): ILM – inner limiting membrane; RNFL – retinal nerve fiber layer; GCL – ganglion cell layer; IPL – inner plexiform layer; INL – inner nuclear layer; OPL – outer plexiform layer; HFL – Henle’s fiber layer; ONL – outer nuclear layer; ELM – external limiting membrane; IS/OS – inner segment/outer segment junction; OS – outer segments; COST/RPE – cone outer segment tips/retinal pigment epithelium; ICH – inner choroid; OCH – outer choroid. (c) Schematics of the three-channel illumination (equilateral triangle geometry). (d) Beam configuration on the 2D-MEMS scanner (in the equilateral triangle geometry all three beams are positioned off-pivot).

Due to OCT’s ability of non-invasively providing histology-like tomograms, human retinal imaging – where OCT originates from – was revolutionized [22]. The retina, considered one of the major targets for OCT imaging, is composed of several neuronal layers interconnected by synapses. Its layered structure and the corresponding intensity changes in depth in OCT image data (cf. Fig. 1(b)

) have been studied extensively [22]. Some layers – such as the retinal nerve fiber layer (RNFL), Henle’s fiber layer (HFL), the inner segment/outer segment junction (IS/OS) or the cone outer segment tips (COST; sometimes also labeled end tips of the photoreceptors (ETPR) or posterior tips of outer segments (PTOS) in the literature) – are known to exhibit angle-dependent optical reflectivity properties. Directional OCT is a technique to contrast directionally reflective structures by recording images using an intent angle alteration of the incident beam [23].

Previous literature supports the assumption that changes in RNFL reflectivity occur prior to thinning of the RNFL in retinas suffering from optic nerve head (ONH) diseases, such as glaucoma [24, 25]. Some studies indicate that an alteration of axonal ultrastructure – which likely holds responsible for the nature of RNFL reflectivity – might precede the eventual loss of axons [26–28]. However, the reflectivity of the RNFL is also known to be strongly directional. This directionality, which was first reported in toads, shows strong similarities to light being scattered by cylindrical structures [29]. Since it appears that an alteration in axonal ultrastructure, and hence a change in RNFL reflectivity seems to be a more reliable indicator of early glaucomatous damage than a change in RFNL thickness [24–28], it is of major importance to account for the directional scattering behavior of the RNFL in reflectivity measurements [30].

Similar to the RNFL, HFL is composed of long cylindrical axons (namely those of the photoreceptors (PR)) and Müller cells, which run obliquely from their nuclei towards their synapse with inner nuclear cells. HFL thickness varies between subjects and by eccentricity from the fovea (maximum thickness at ~1 mm eccentricity from the foveal pit) [31, 32]. Because of its oblique fiber orientation and its highly directional reflectivity, conventional OCT is not able to visualize HFL since no reflectivity change at the HFL/ONL interface can be observed as long as the beam entry position is centered at the subject’s pupil. By lateral displacement of the beam entry position, single sectors of HFL were first intentionally visualized in 2011 [31, 33]. To contrast and segment parts of HFL, several OCT scans at different beam entry positions have to be acquired consecutively and registered afterwards [34]. Measuring HFL thickness is of indirect but vital importance for ONL thickness measurements, since the latter are strongly overestimated if HFL is not taken into account. ONL thickness is considered a key biomarker in differentiation of normal aging changes from degenerative diseases [23, 35]. Apart from ONL thickness measurements, the reflectivity properties of HFL might additionally be of interest as they may enable valuable insight into pathophysiological processes.

In OCT image data, the IS/OS-COST complex is part of what is called the outer retinal bands. Coming from the inner retina, the five hyperreflective bands (anatomically resembling the PR and retinal pigment epithelium (RPE) layer) are the external limiting membrane (ELM), the IS/OS, the COST, the rods outer segment tips (ROST) and RPE [36, 37] – cf. Fig. 1(b). Most commonly ROST and RPE cannot be resolved as single bands, but combine to a highly reflective broadened complex. The wave-guiding property of PRs is known as the (psychophysical) Stiles-Crawford effect (SCE) [38], which in terms of retinal reflection leads to its optical analogon – the optical SCE (OSCE). For reasons of increased light coupling efficiency of the incident radiation, the central axes of the PRs point towards the pupil center in the unaberrated eye [39]. Due to this orientation, sensitivity to intraocular stray light is decreased while on the other hand sensitivity to light incident from the pupil center is increased. In addition, the eye’s resolution is enhanced [40]. However, not only the orientation, but also the relation between beam spot size and diameter of the respective PR seems to influence its directional reflectivity [41]. Concerning OCT, the IS/OS and the COST were previously found to be highly directionally reflective structures and thus very sensitive to the beam entry position in the pupil plane [42]. It seems the directionality in terms of reflectivity of the IS/OS-COST complex is more pronounced at peripheral regions in comparison to foveal regions [43–46]. However, without doubt further investigations to better understand the directional reflectivity of the IS/OS-COST complex are required. Nevertheless, already very small irregularities in tissue composition and arrangement will display as hyper- or hyporeflective areas, which might aid in early diagnosis of ophthalmic diseases [46, 47].

In this paper, we merge the idea of directional OCT with the concept of multi-channel OCT. Multi-directional OCT [48] enables more detailed investigations of materials and tissues exhibiting directional optical reflectivity from different angles simultaneously. This work focuses on investigation of angle-dependent reflectivity changes of distinct retinal regions and layers (RNFL, HFL, IS/OS, COST). We demonstrate the potential of the technique by extracting additional morphological information out of three simultaneously acquired reflectivity scans at the retinal locations of and surrounding the ONH (peripapillary region) and the macular region in the eyes of healthy volunteers.

## 2. Methods

### 2.1 Experimental setup

A three-beam spectral domain (SD-) OCT prototype – initially developed for total retinal blood flow (TRBF) measurements using DOCT and discussed in greater detail elsewhere [20, 49, 50] – was employed for our multi-directional OCT investigations. The system’s three channels enable simultaneous probing of a mutual sample location from three linearly independent orientations. In short, the setup features three superluminescent diode (SLD) sources (*λ_0_* = 840 nm; *Δλ* = 50 nm), a joint bulk optics Michelson interferometer as well as three identical spectrometer units. At 40 kHz line-scan frequency and a beam power of 233 μW per beam (thereby satisfying the maximum permissible exposure (MPE) limits regarding the laser safety requirements [51]) the system’s sensitivity was measured to be ~93.5 dB per channel. The three collimated sample arm beams were aligned parallel to each other and arranged according to the corners of an equilateral triangle (beam separation distances at the pupil plane: ~2.5 mm) – cf. Fig. 1(c). Considering beam diameters of ~0.8 mm (corresponding to a transverse resolution of ~25 μm for retinal imaging), the present beam geometry allowed for penetration of the undilated human pupil at low light conditions. The three beams were eventually focused onto a mutual spot by the refractive optics of the human eye – cf. Fig. 1(c). The inclination between the individual sampling beams was ~3.5° for an eye of standard length.

### 2.2 Scanning parameters and measurement protocol

For 2D scanning of the three sampling beams a dual-axis gimbal-less MEMS mirror (Mirrorcle Technologies, Inc.; diameter 3.6 mm; mechanical tilt angle: −6.5° to 6.5°) was used. The three collimated beams were aligned in an equilateral triangle configuration (beam separation distances 1.6 mm) and incident on the MEMS scanner as depicted in Fig. 1(d). This geometry did not permit pivot scanning for any of the respective beams.

Different scanning parameters in terms of numbers of acquired A-/B-scans, scanning location as well as scanning patterns were used in this study. The two retinal regions of the ONH and the macula were considered the most promising scanning locations, since the focus of this study was on imaging the RNFL, HFL and the PR layer. For the peripapillary region a circumpapillary (CP) scanning pattern – cf. Fig. 1(a) – was applied, consisting of 6144 A-scans per circular B-scan (circle diameter: corresponding to a field of view (FoV) of ~10°). For the macular region a linear as well as a raster scanning pattern was applied – cf. Fig. 1(a). The linear pattern consisted of 8192 A-scans per B-scan, while the raster pattern consisted of 2048 A-scans × 250 B-scans.

The in vivo measurements on three healthy volunteers (age range: 27-30) performed in this study were carried out after informed consent was obtained. The study was approved by the institutional ethics committee and was in agreement with the tenets of the Declaration of Helsinki.

A standard head rest, adjustable in x-, y- and z-direction, was used in order to align the subject’s pupil one focal distance from the second telescope lens. At this pupil position, the scanning movement of the three sampling beams was minimal. For correct positioning of the pupil, a simple low-cost video camera (sensitive at *λ_0_* = 840 nm) was used. To adjust the imaging location on the retina, a fixation target was displayed to the subject’s contralateral eye.

### 2.3 Data processing

Standard SD-OCT post processing – mean spectrum subtraction, *λ*-*k*-resampling, numerical dispersion compensation, FFT – was performed on the acquired raw data using custom-developed LabView code. The more advanced post-processing steps – including image registration, retinal layer segmentation, color-coding, quantitative feature evaluation, intensity normalization (to the RPE) and graph smoothing (methods: lowess, loess) – were carried out in ImageJ and Matlab.

Image registration (ImageJ: StackReg – rigid body) among the three simultaneously acquired images needed to be performed mainly in order to eliminate depth offsets between the three channels (the individual images are acquired at slightly different imaging depths due to minor mismatches in the lengths of the three respective sample arms).

To enable quantification of imaging results as well as registration of B-scans within a 3D data volume, automated retinal layer segmentation was performed. The applied algorithm was a graph based approach adapted from [52, 53]. To quantify directional intensity changes in the IS/OS-COST complex a 20-depth-pixel deep window (~70 μm) was evaluated regarding its mean intensity per A-scan. The window was determined by first segmenting the RPE and then shifting its location ten pixels anterior to exclude the directionally invariant intensity signal of the RPE itself. To quantify changes in the RNFL, a 15-depth-pixel deep window (~53 μm) was evaluated. The window was determined by first segmenting the ILM and then shifting its location three pixels posterior to exclude specular reflections at the transition zone from vitreous to retina. To enable 3D display of the acquired data sets, registration based on the automated segmentation was performed. Thereby, the retinal surface (ILM) of the image stack was segmented B-scan wise and flattening was applied. Then the data cubes were fused, before reshaping the surface to the shape of channel-1 to regain a realistic foveal shape.

**Fig. 2:**
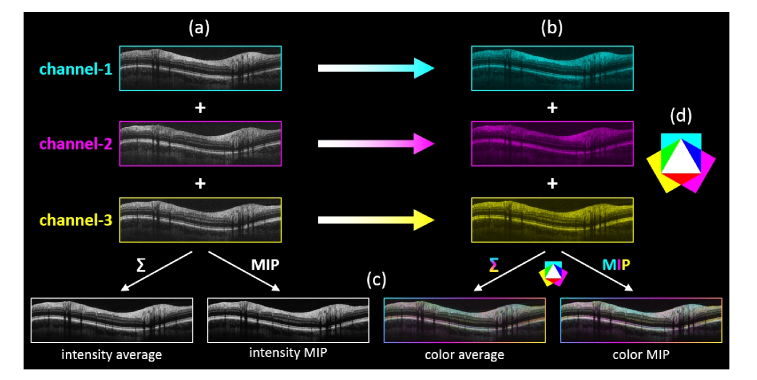
Flowchart diagram of image analysis for revealing directional contrast among the three simultaneously acquired intensity B-scans. (a) Single-frame registered B-scans of the three channels. (b) Color coded B-scans of (a). (c) Intensity/color averaged and intensity/color maximum intensity projection (MIP) of the three respective fused B-scans. (d) Additive color scheme of primary and secondary colors.

In order to provide a decent understanding of the figures presented in the following sections, a flowchart diagram of the image analysis performed for revealing directional contrast among the three simultaneously acquired intensity scans is depicted in Fig. 2

.

## 3. Results

### 3.1 Investigation of the directional scattering properties of the IS/OS-COST complex

In Fig. 3

**Fig. 3:**
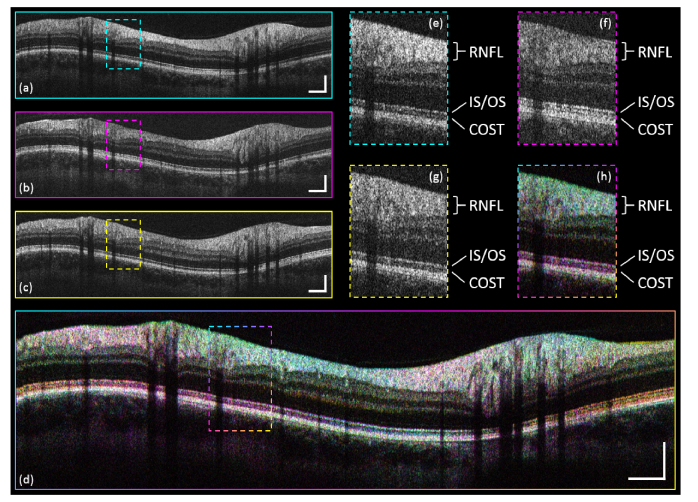
CP intensity B-scans of the ONH region. (a) Channel-1 (cyan). (b) Channel-2 (magenta). (c) Channel-3 (yellow). (d) Color averaged image of the three channels. (e)-(h) Zoom-ins of the indicated respective ROI. Scale bars: 0.5 mm (horizontally), 0.2 mm (vertically).

CP intensity B-scan imaging results of a healthy volunteer are shown. Figures 3(a)-3(c) depict single-frame scans of each of the three simultaneously acquired channels. Figure 3(d) shows a registered color averaged image generated from Figs. 3(a)-3(c). Here, the colors used – cyan, magenta, yellow – add up to the color white (or, in dependence of the summed intensity, actually a shade of gray) if the respective pixel intensities of all three channels are equal. For pixels of strongly varying intensity values color offsets depending on the dominating channel(s) and indicating directional reflectivity will display. This can either result in a pure color offset (if one channel shows significantly higher intensity than the other two) or in any intermediate color offset by combination of the respective colors. Image areas where pixel intensities are generally more uniform among the three channels (e.g. the RPE) will not exhibit any color offset. Figures 3(e)-3(h) depict zoom-ins of the region-of-interest (ROI) indicated in Figs. 3(a)-3(d).

In Figs. 3(a)-3(c), intensity variations along the CP B-scan which can be attributed to directional reflectivity are noticeable (in particular in the RNFL and the IS/OS-COST complex). These intensity variations differ by location among the channels. In the zoom-ins – Figs. 3(e)-3(g) – the intensity differences are easier to be observed. The RNFL shows unequal intensity distribution but the differences in the IS/OS-COST complex are even more pronounced. The COST-layer shows up prominently only in Fig. 3(f) while in Fig. 3(e) and Fig. 3(g) it is almost invisible. Also the IS/OS-layer is more pronounced in Fig. 3(f).

The color averaged image – Fig. 3(d) – offers the possibility to directly distinguish areas of directional hyper- from those of hypo-reflectivity, within one image. Besides color offsets due to specular reflections at the inner limiting membrane (ILM), the most distinct color offsets are detected in the RNFL and IS/OS-COST complex. In the IS/OS-COST complex three color sectors can be observed (yellow to magenta to cyan to yellow again). These sectors are roughly separated by ~120° in the CP B-scan which is a direct consequence of the sampling beam geometry (in the equilateral triangle configuration the separation angle between two beams is also 120°). The three sectors represent one intensity oscillation per channel along one CP scan. For the RNFL the color offsets are not as pronounced as for the IS/OS-COST complex, however, slight color changes along the CP scan are noticeable. Here two color oscillations (yellow to magenta to cyan to yellow to magenta to cyan to yellow again) are faintly visible, which might be attributed to the already reported cylindrical scattering behavior of the RNFL [29].

Figure 4

**Fig. 4:**
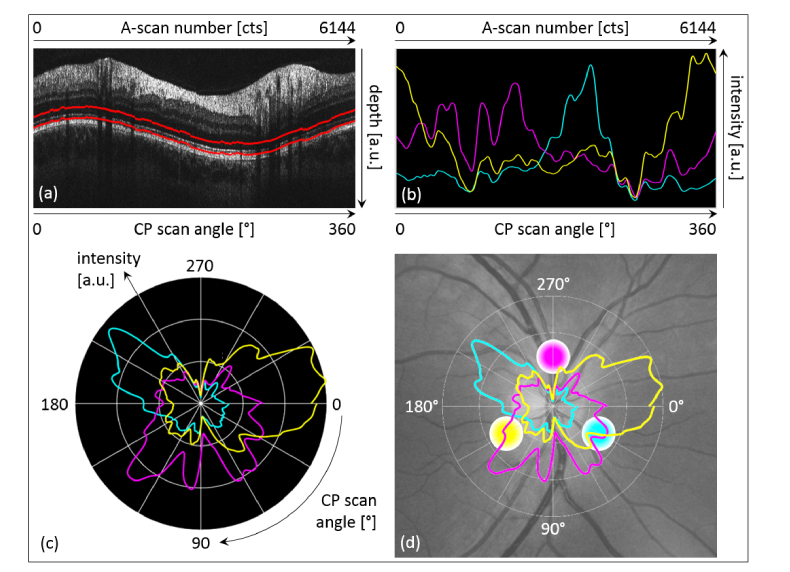
Quantitative evaluation of the IS/OS-COST complex. (a) Representative CP B-scan indicating the evaluation window in between the red lines (20 depth-pixels or ~70 μm). (b) Normalized and smoothed intensity distribution within the evaluation window as a function of the azimuth angle (channel-1: cyan; channel-2: magenta; channel-3: yellow). (c) Polar plot of (b): intensity (radius) as a function of the azimuth angle. (d) Color fundus photo (grayscale) overlay of (c) and indication of the respective equilateral triangle beam geometry.

describes the quantitative evaluation of the aforementioned observations in the IS/OS-COST complex using automated layer segmentation. Figure 4(a) depicts a representative CP B-scan including the lower and upper margin of the evaluation window (red lines). The intensity distribution in the evaluation window along the three simultaneously acquired representative CP B-scans (ten consecutive CP B-scans evaluated per channel) is presented in Fig. 4(b) as a function of the azimuth angle (data was normalized and smoothed). Even though vessel shadowing obviously obstructs parts of the evaluation window (cf. vessel shadows reducing or eliminating the corresponding intensity signal in Fig. 4(a)), three sectors (one per channel) can be clearly distinguished from each other by their peak intensities. To facilitate a more intuitive understanding of this observation, a polar plot (intensity (radius) as a function of azimuth angle) is provided – cf. Fig. 4(c). Here, the three sectors (roughly separated by ~120°) can be identified easily. Figure 4(d) comprises an overlay of the polar plot onto a color fundus photo of the studied eye displayed in grayscale. This overlay demonstrates that intensity signal reduction in some areas of the graph can be attributed to vessel shadowing. In addition, the equilateral triangle beam geometry is also displayed in order to confirm the characteristic directional backscattering behavior of the photoreceptors.

**Fig. 5:**
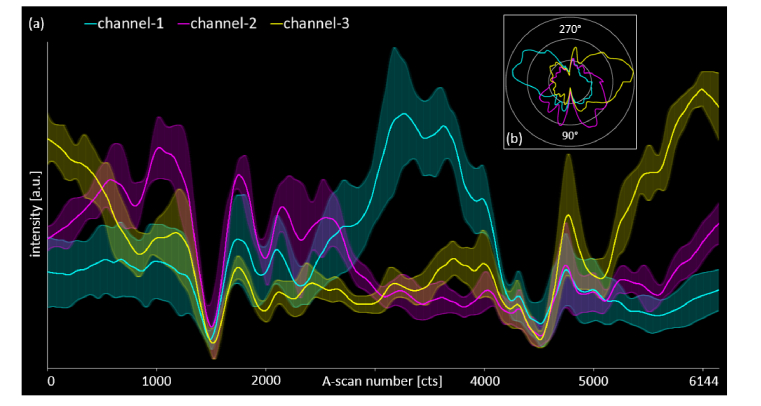
Investigation of the repeatability of the quantitative evaluation of the IS/OS-COST complex in one eye of a healthy volunteer. (a) Mean and standard deviation (shaded area indicates ± one standard deviation) of five measurements (acquired within two consecutive days) per channel (channel-1: cyan; channel-2: magenta; channel-3: yellow). Normalized intensity as a function of the A-scan number (or the respective azimuth angle). (b) Polar plot of the mean data of (a): intensity (radius) as a function of the azimuth angle.

To investigate the repeatability of these results, five measurements of the same eye were acquired within two consecutive days (minimum time duration in between two measurements: two hours). The results are displayed in Fig. 5

. The normalized intensity mean plus respective standard deviations of the five data recordings per channel are plotted as a function of A-scan number of the respective CP B-scan in Fig. 5(a). As for the single measurement before, the three ~120°-separated color sectors can be identified easily, although vessel shadowing again severely obstructs especially the magenta sector represented by channel-2. In Fig. 5(b) the polar plot of the mean values is depicted in order to allow a comparison to the results from Fig. 4. Although subject alignment (eye positioning) and subject fixation – which are never perfectly identical for subsequent in vivo measurements – definitely have an impact on the outcome of a multi-directional OCT measurement, the repeatability evaluation suggests our method to reliably identify directional reflectivity of the IS/OS-COST complex.

**Fig. 6:**
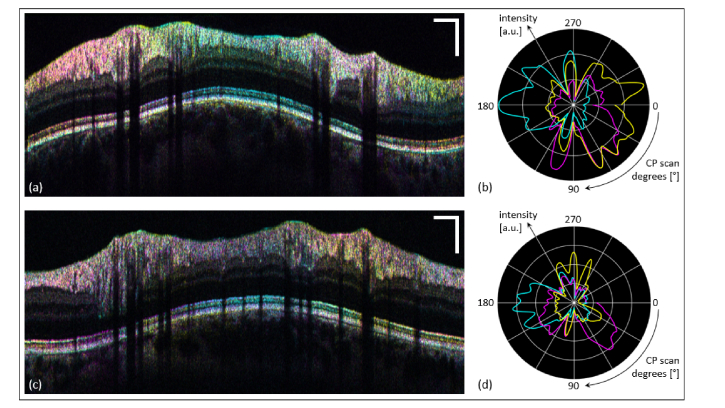
Investigation of the variability among subjects of multi-directional OCT findings in two additional eyes of two healthy volunteers. (a), (c) Color averaged images of two eyes of two subjects. (b), (d) Respective normalized and smoothed polar plots of the intensity distribution within the evaluation window as a function of the azimuth angle (channel-1: cyan; channel-2: magenta; channel-3: yellow). Scale bars: 0.5 mm (horizontally), 0.2 mm (vertically).

The variability among subjects was evaluated by performing measurements on two more eyes of two healthy volunteers. The obtained results are depicted in Fig. 6

. Although a predominant three sector partitioning can be observed for all three eyes, the inter-eye variation proves much stronger than the repeatability variance of the same eye. Since in total only three eyes were evaluated, the inter-subject variability was not studied in greater detail here. More extensive investigations, possibly also considering pathologic alterations of PR alignment, might provide better understanding of these variations.

### 3.2 Investigation of the directional scattering properties of the RNFL

Figure 7

**Fig. 7:**
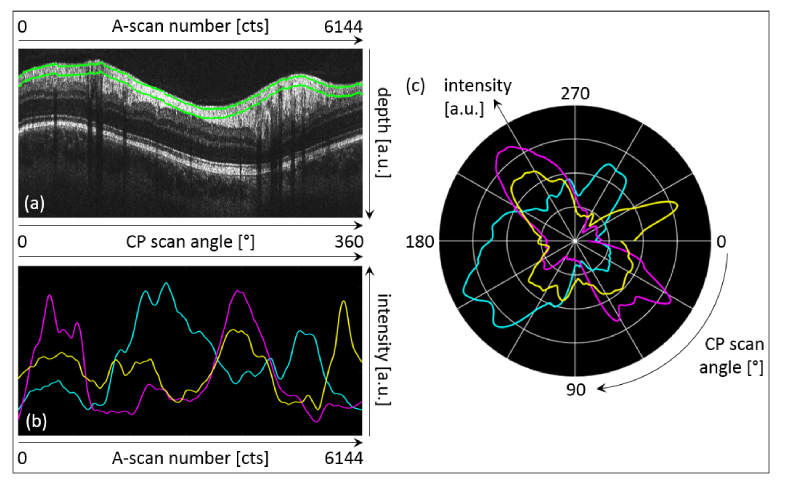
Quantitative evaluation of the RNFL. (a) Representative CP B-scan indicating the evaluation window in between the green lines (15 depth-pixels or ~53 μm). (b) Normalized and smoothed intensity distribution within the evaluation window as a function of the azimuth angle (channel-1: cyan; channel-2: magenta; channel-3: yellow). (c) Polar plot of (b): intensity (radius) as a function of the azimuth angle.

demonstrates the quantitative evaluation of the directional intensity changes in the RNFL using automated layer segmentation. Figure 7(a) depicts a representative CP B-scan including the lower and upper margin of the evaluation window (green lines). Again – similar to Fig. 4(b) – the intensity distribution in the evaluation window along the three simultaneously acquired CP B-scans (ten consecutive CP B-scans evaluated per channel) is presented in Fig. 7(b) as a function of the azimuth angle (data was normalized and smoothed). In this case the evaluation window is not obstructed by vessel shadowing but by the vessels themselves. Even if the interpretation of the observed graphs is not as straight forward as for the IS/OS-COST complex, more or less six sectors or two color oscillations (yellow to magenta to cyan to yellow to magenta to cyan to yellow again) can be observed. Figure 7(c) provides the respective polar plot of Fig. 7(b). At least the two cyan and the two magenta sectors are separated by about 180° which suggests that the previously reported cylindrical scattering behavior of the RNFL [29] might account for this observation.

### 3.3 Investigation of the directional scattering properties of HFL

**Fig. 8:**
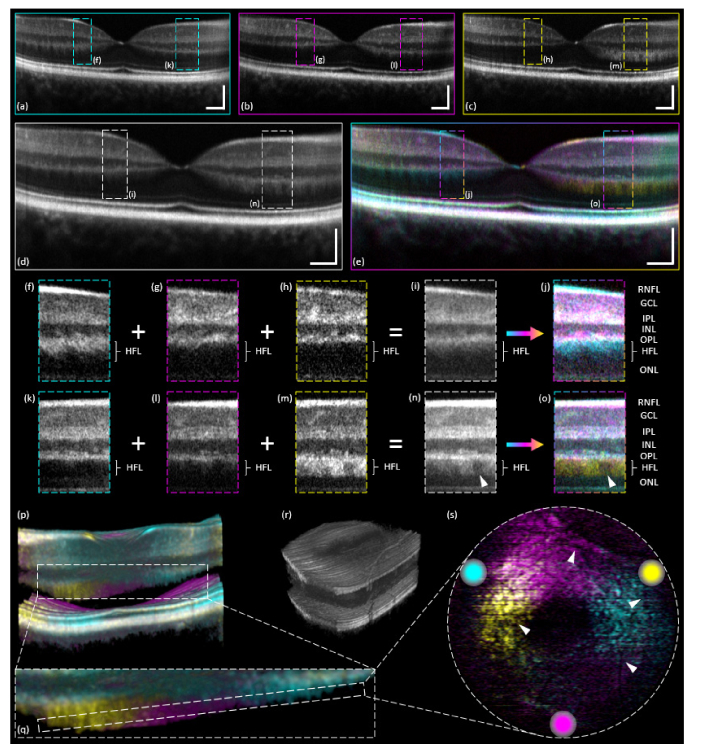
Linear ((a)-(o); 16-times averaged) and volumetric ((p)-(s); single-frame) intensity macular imaging results. (a) Channel-1 (cyan). (b) Channel-2 (magenta). (c) Channel-3 (yellow). (d) Intensity average of the three channels. (e) Color averaged image of the three channels. (f)-(o) Zoom-ins of the indicated respective ROIs. (p) Cross-sectional view of a 3D rendering of a color MIP near the foveal pit. (q) Zoom-in of the indicated ROI in (p) to better visualize HFL. (r) 3D rendering of the volumetric data cube (channel-2). (s) Visualization of HFL in a color MIP en-face projection including indication of the respective equilateral triangle beam geometry. Scale bars: 0.5 mm (horizontally), 0.2 mm (vertically).

First intentional visualization of HFL in OCT intensity scans [31, 33] initialized more detailed OCT investigations on directional scattering properties of retinal tissue, which today can be summarized under the term directional OCT. Thus, obviously, HFL was considered a target area for multi-directional OCT. HFL can only be visualized in OCT intensity scans if the incident light and the direction of alignment of Henle’s fibers are orientated perpendicular to one another. The more the incident angle deviates from perpendicularity, the less signal from HFL is detected. Since the equilateral triangle beam geometry (cf. Fig. 1(c)) of the instrument in use was not easily adjustable without complicated realignment and calibration procedures of the whole system, the incident angles onto the retinal tissue were fixed. The triangle beam geometry only permitted slight lateral subject adjustments (in dependence of the size of the pupil aperture of the subject) to not have any of the beams blocked by the iris. Any further angular adjustment of the three incident beams was thus severely limited. However, even though the angles of incidence of the present beam geometry did not match the requirements of perpendicular incidence perfectly, initial multi-directional OCT investigations to visualize HFL could be performed and respective image data are presented in Fig. 8

.

Figures 8(a)-8(o) depict linear macular B-scans through the foveal pit. The three simultaneously acquired intensity B-scans – cf. Figs. 8(a)-8(c) – were averaged 16-times. Figure 8(d) depicts an intensity average while Fig. 8(e) presents a color averaged image of Figs. 8(a)-8(c). Figures 8(f)-8(o) are zoom-ins of the indicated ROIs of Figs. 8(a)-(e).

The 2D retinal cross-sections are intentionally tilted slightly to the right. This was done in order to clearly visualize HFL at least on one – in this case the right – side of the foveal pit. The fixed triangle beam geometry did not enable clear visualization of the HFL on both left and right side simultaneously.

Close examination enables the observation of HFL already in Figs. 8(a)-8(c). Parts of HFL are visible on the left hand side of the foveal pit in Fig. 8(a) and especially pronounced on the right hand side of the foveal pit in Fig. 8(c). Left of the foveal pit in Fig. 8(c), a very sharp borderline in between OPL and HFL is detectable and can be explained by the total absence of backscattering signal from HFL. Even though HFL can be distinguished by the trained observer from the OPL in Fig. 8(d) on both sides of the foveal pit, the fused color intensity image (Fig. 8(e)) better contrasts HFL (left of the foveal pit: cyan; right of the foveal pit: yellow). In the zoom-ins of two ROIs (Figs. 8(f)-8(o)) the contrast was adjusted in order to highlight the visual absence (Figs. 8(h), 8(k)) as well as the visual presence (Figs. 8(f), 8(m)) of HFL (including intermediate cases in Figs. 8(g), 8(l)). Even though all other retinal layers can be distinguished from each other – as labeled in Figs. 8(j), 8(o) – HFL sticks out due to its cyan (Fig. 8(j)) or yellow (Fig. 8(o)) color offset.

To further investigate HFL, volumetric macular scans using a raster scan pattern were acquired. Figures 8(p)-8(s) present 3D multi-directional OCT image data. Figure 8(p) presents a cross-section of a 3D rendering of a color MIP near the foveal pit, depicting parts of HFL in each of the respective colors. For better visualization (due to weaker signal intensity of HFL) a color MIP was favored here in comparison to the color averaged images of the previous sections. Also a zoom-in of the indicated ROI is provided in Fig. 8(q). Here, the lower boundary of HFL was segmented manually and all pixels below it were set to black. In Fig. 8(s) HFL is visualized via a color en-face MIP (slice thickness: ~25 μm) of the area indicated in Fig. 8(q). The en-face map shows three color sectors of HFL which are arranged not perfectly in a concentric ring shape around the foveal pit. This is caused by a slight off-axis tilt (same reasons as aforementioned for the linear scans) of the acquired volume scan, which consequently influences the backscattering signal from the HFL. Nevertheless, a major part of HFL could be visualized in 3D within one acquisition using multi-directional OCT.

### 3.4 Compensation of directional reflectivity

**Fig. 9:**
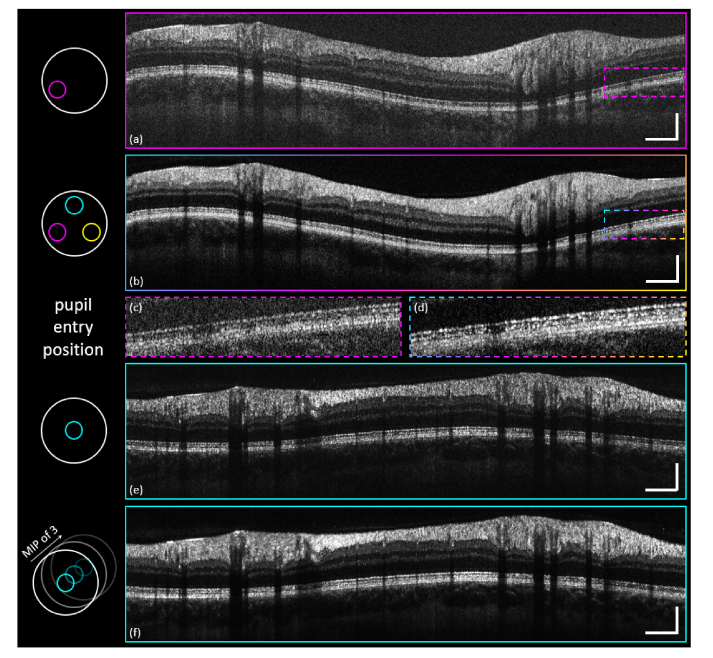
Compensation of directional reflectivity and comparison of pupil entry position. (a) Single-frame CP intensity B-scan of the off-centered channel-2. (b) Three-channel off-center CP intensity B-scan MIP. (c), (d) Indicated ROI zoom-ins of the PR-layer. (e) Single-frame CP intensity B-scan of the pupil-centered channel-1. (f) MIP of a CP intensity B-scan of the three-frame averaged pupil centered channel-1. Scale bars: 0.5 mm (horizontally), 0.2 mm (vertically).

Using our multi-directional approach, we are not just able to highlight differences in the simultaneously acquired intensity data sets, but we can also use the additional information to reconstruct images that are insensitive to intensity fluctuations caused by directionally reflective structures. This compensation of directional reflectivity enables a close to constant intensity distribution along the whole cross-sectional scan for all retinal layers. Figure 9

demonstrates that a simple maximum intensity projection (MIP) of three simultaneously acquired, registered and fused CP B-scans allows for this compensation of directional reflectivity. In comparison to the single-channel CP B-scan (Fig. 9(a); channel-2), the MIP of the fused intensity scans (Fig. 9(b)) does not show obvious intensity variations along the RNFL or the IS/OS-COST complex. Figures 9(c), 9(d) depict zoom-ins of the indicated ROI at the PR-layer which highlights the intensity difference of the COST-layer.

To provide a better comparison to standard OCT imaging (the beams entering the eye for the acquired CP intensity B-scans – Fig. 9(a), 9(b) – were off-center with regards to the subject’s pupil) a CP scan, where channel-1 was aligned to penetrate the pupil at its center, was obtained. The other two channels were consequently partially blocked by the iris. Figure 9(e) depicts a single-frame image, while Fig. 9(f) depicts a CP intensity B-scan MIP averaged over three consecutive scans to enable an even comparison in terms of SNR to Fig. 9(b).

Although acquired from the same eye, the two compared CP B-scans do not match perfectly in terms of shape or vessel profile. However, a comparison in terms of intensity distribution in the IS/OS-COST complex along the scan is still appropriate. By visual comparison of Figs. 9(b) and 9(f) a more homogenous IS/OS-COST complex can be identified in Fig. 9(b) (also true for the RNFL). It seems that even a pupil centered acquisition does not enable a perfectly homogenous backscattering signal from the referred retinal layers (which is probably due to misalignment of the PRs caused by optical aberrations of the eye). Especially on the far right of Fig. 9(e), 9(f) the signal from the COST region is clearly reduced. For the ophthalmologist, there is no way to distinguish whether the signal reduction is due to directional reflectivity or pathology. On the other hand, Fig. 9(b) gives a clear indication that the reduction of signal is caused by directional reflectivity only.

To summarize, in terms of visualization of directionally reflective retinal tissue multi-directional OCT provides better imaging results in comparison to a conventional pupil centered scan. Even though the single-channel off-center CP B-scans show stronger signal variation along one scan in comparison to a pupil centered one, a MIP of the three simultaneously recorded images allows for compensation of directional reflectivity and thus enables differentiation from image features caused by pathology.

### 3.5 Speckle reduction

**Fig. 10:**
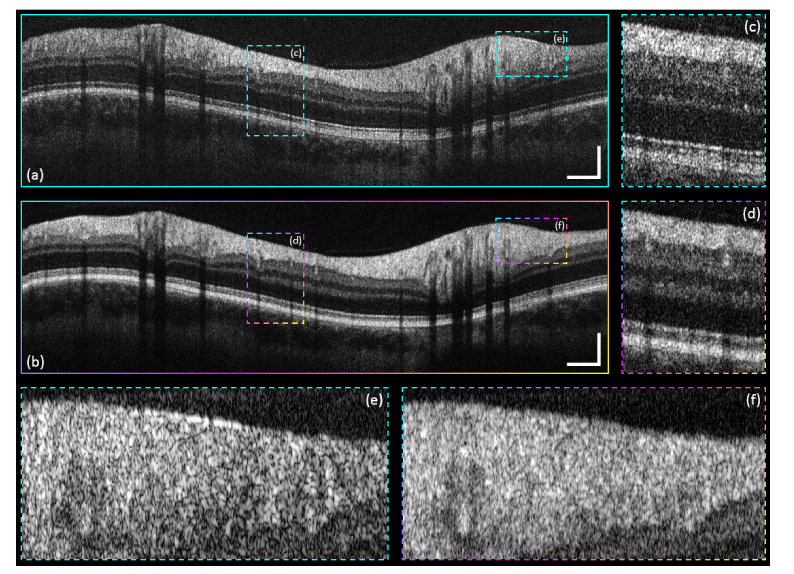
Speckle reduction comparison. (a) Single-frame CP intensity B-scan of channel-1 (a). (b) Three-channel-averaged CP intensity B-scan. (c), (d) Indicated ROI-1 zoom-in on the entire retinal cross-section. (e), (f) Indicated ROI-2 zoom-in on the RNFL. Scale bars: 0.5 mm (horizontally), 0.2 mm (vertically).

Speckle reduction through averaging of the simultaneously acquired intensity scans presents another advantage coming along using multi-channel OCT [4]. In Fig. 10

the speckle reduction capabilities of our imaging technique are presented. Besides a comparison of the entire CP B-scan – Fig. 10(a): single-frame CP B-scan of channel-1; Fig. 10(b): intensity average of three simultaneously acquired, registered and fused CP B-scans – the marked ROIs – Figs. 10(c)-10(f) – provide a better indication of how speckle can be reduced by threefold averaging.

To quantify image quality differences, well-established speckle reduction performance metrics such as signal-to-noise ratio (SNR), contrast-to-noise ratio (CNR) and equivalent number of looks (ENL) [54] were used over five ROIs (each 100 × 50 pixels) per image. SNR, CNR and ENL are defined as

$$SNR=10\times \log_{10}\left(\frac{\mu_{s.lin}^{2}}{\sigma_{b,lin}^{2}}\right),$$ (1)

$$CNR=\frac{\mu_{s,\log}-\mu_{b,\log}}{\sqrt{\sigma_{s,\log}^{2}+\sigma_{b,\log}^{2}}},$$ (2)

$$ENL=\frac{\mu_{s,\log}^{2}}{\sigma_{s,\log}^{2}},$$ (3)

where *μ_s,lin_* represents the mean of intensity of a ROI within a homogenous signal part of the image (in this case within the RNFL) and *σ_b,lin_* represents the standard deviation of intensity of a ROI within the background noise part of the image – both calculated on linear OCT data. *μ_s,log_* and *σ_s,log_* represent the mean and standard deviation of intensity of a ROI within a homogenous signal part of the image. *μ_b,log_* and *σ_b,log_* represent mean and standard deviation of intensity of a ROI within the background noise part of the image. The latter four values are calculated on logarithmic OCT data. In Table 1

**Table 1:** Speckle reduction performance metrics SNR, CNR and ENL for the CP scans depicted in Fig. 10 averaged over five ROIs per image (homogenous signal part from within the RNFL). The single-frame CP B-scans of the three individual channels were evaluated and the respective mean values were compared to the values obtained from the intensity averaged CP B-scan.

	**SNR [dB]**	**CNR**	**ENL**
channel-1	29.6	3.2	271.6
channel-2	25.6	2.6	293.4
channel-3	28.3	3.0	272.7
mean	**27.8**	**2.9**	**279.2**
intensity average	**32.9**	**5.0**	**794.2**

the respective metrics are summarized.

The SNR of the intensity average is improved by ~5 dB in comparison to the mean SNR of the single-frame images of the three channels. The CNR – which measures the contrast between a ROI of image feature and a ROI of background noise – is improved by a factor of ~1.7, whereas the ENL – which measures the smoothness of a speckle-corrupted homogenous ROI – is improved by a factor of ~2.8.

## 4. Discussion

Our multi-directional OCT approach enables in-depth investigations on the reflectivity of directionally scattering structures. In this manuscript, the multi-channel technique was demonstrated for in vivo retinal imaging in the eyes of healthy human volunteers. However, in principle, the concept can be extended to a wide variety of samples beyond the field of ophthalmology or biomedical optics.

In comparison to directional OCT [23], multi-directional OCT enables to visualize, contrast and segment directionally reflective structures within a single data acquisition. Moreover, retinal directional OCT makes use of multiple B-scans acquired consecutively at different pupil entry positions of the sampling beam – which implies repeated subject alignment – to do so [55]. The re-alignment makes it more challenging in terms of imaging exactly the same retinal section, whereas multi-directional OCT offers the advantage of simultaneous acquisition of all needed information. Thus, no sophisticated image registration algorithms are needed. Additionally, the differing pupil entry positions used in directional OCT implicate a tilting of the retinal surface in the cross-sectional B-scans which particularly in SD-OCT systems – which often suffer from a more severe sensitivity roll-off – might obscure measurement results. Our multi-directional OCT setup, on the other hand, enables acquisition of three untilted retinal cross-sections at the same time.

The reason for the untilted retinal cross-sections provided by our instrument has neither been discussed nor has this property been used for directional OCT. Since our three sampling beams enter the pupil off-center – according to the principles of directional OCT [31] – three tilted cross-sectional scans should be observed. That this is not the case can be explained by the off-pivot position of each of the three beams on the 2D-MEMS scanning mirror – cf. Fig. 1(d). Off-pivot scanning eventually compensates for the path-length difference responsible for tilting the retinal cross-section, which is introduced by an off-center pupil entry position. This can be comprehended by considering the case of a single sampling beam with large diameter that hits the scanning mirror with its central ray at the pivot point. All peripheral rays of the beam are consequently scanned off-pivot. Nevertheless, the entire beam will generate an untilted retinal cross-section, i. e. all the peripheral rays that are scanned off-pivot and enter the pupil off-center will contribute to the untilted cross section. Our three sampling beams can therefore be regarded as individual components (sub-beamlets) of a large diameter beam and thus generate untilted retinal B-scans. Hence, directional OCT would in principle also allow for acquisition of untilted retinal cross-sections as long as the sampling beam is set to a corresponding off-pivot scanning position. However, since most directional OCT studies have been based on commercial instruments, off-pivot scanning could not have been realized straightforward.

The mutual focus spot condition, as mentioned in section ‘2.1’, only applies for an ideal imaging scenario. In practice, the anatomical features of each individual eye influence this condition. A perfect overlap of all three beams for every eye, every retinal location and every ROI is not feasible, small lateral offsets are unavoidable without the use of adaptive optics. Nevertheless, for imaging mostly horizontally oriented structures such as the RNFL, HFL or the PR layer, the deviation from the mutual focus spot condition was found to be negligible. Otherwise the shapes of the three respective OCT intensity scans of the three channels would not coincide with the observed high degree. However, a potential lateral offset will result in discrepancies among the three simultaneously recorded scans when imaging more vertically oriented retinal structures, such as the fiber bundles of the RNFL descending into the depression of the ONH, through the lamina cribrosa. Here, the mutual focus spot condition might be violated to a degree which would necessitate additional feedback controlled alignment measures such as adaptive optics for correction [56].

Another interesting finding concerns the appearance of areas of decreased signal intensity within the HFL – a retinal layer that in histology appears smooth and homogeneous [31]. Although 16-fold averaging was applied, a rather inhomogeneous intensity distribution is observed – cf. Fig. 8(n), 8(o) (white arrowheads). The same appearance can also be found in numerous previously published directional OCT B-scans [23, 31, 33, 34] (without being discussed in the referred papers). Moreover, also in conventional OCT B-scans (pupil centered beam entry) a ragged border area between OPL and HFL shows up frequently. This observation might originate from the same phenomenon. A different visualization of this finding can also be observed in the en-face projection of the 3D data volume – cf. Fig. 8(s) (white arrowheads). Here, some kind of network structure showing decreased intensity signal is noticeable within HFL. Even though the origin of this effect remains unclear, two explanations – vessel shadowing and a sudden change in fiber orientation – can be discarded. Firstly, vessel shadowing would lead to reduced signal also in posterior layers. Secondly, sudden changes in fiber orientation are not in accordance with histologic findings [31]. Further investigations are needed to clarify the observed characteristic.

Speckle reduction presents another benefit of multi-directional OCT [4]. Even though only a third of the possible illumination power permitted by MPE limit was used for each of the three channels [51], subsequent averaging of the acquired B-scans retrieved similar SNR as for single-channel directional OCT. However, the applied threefold angular averaging considerably decreases speckle appearance – cf. section 3.5.

By fusing the intensity information of all three channels, directional reflectivity can be compensated for in a way that the intensity level along the respective B-scan remains rather constant. By application of an MIP the directionally reflective layers show satisfying signal along an entire B-scan – cf. Fig. 9(b). However, even if the directional reflectivity of retinal structures can be compensated for using multi-directional OCT, three separate beams might not be the optimum. More observation orientations might further improve the compensation.

However, our multi-directional OCT setup also suffers from shortcomings. Two intrinsic problems are hardware based and may be corrected for in the future. The first being the non-adjustable beam separation. The highly complex bulk optics Michelson interferometer setup – cf [20, 49, 50]. – requires a complete setup re-alignment after each beam separation adjustment. Nevertheless, an easy adjustable beam separation – meaning adjustable inclination angles of the incident beams on the retina – would mean a considerable expansion of multi-directional OCT towards customizability. Especially in terms of signal detection from HFL in a 2D linear scan trough the foveal pit, the equilateral triangle beam geometry did not permit full visualization on both sides of the fovea simultaneously. Ideally the angular separation between two sampling beams for this scenario should be 180° instead of 120°. Thus, a slight retinal tilt was applied for acquisition of the images presented in Fig. 8. To address this issue a modified sample arm design – e.g. as recently presented in our sequential multi-channel OCT prototype [57] – would needed to be implemented.

The second hardware based shortcoming concerns the scan range of the 2D-MEMS mirror. Limited by the mechanical maximum tilt angle (−6.5° to 6.5°) of the mirror, only a rather small FoV can be obtained. Advancements in 2D-MEMS mirror technology in terms of increased scanning angles would benefit the system and enable wide-field multi-directional OCT investigations.

A potential source of error for ophthalmic multi-directional OCT may be beam vignetting at the subjects pupil. Considering beam separation distances of ~2.5 mm and beam diameters of ~0.8 mm [50] the minimally required pupil aperture was calculated to be ~3.7 mm. Only measurement data from subjects with a pupil aperture diameter larger than that (at low light conditions) can be considered reliable for multi-directional analysis, since otherwise intensity variation along a B-scan might originate from beam vignetting. However, an artificial non-physiological dilation of the pupil using mydriatics would help prevent this problem.

Future retinal multi-directional OCT research might focus on the investigation of major ophthalmic diseases, such as AMD or glaucoma. Since alterations in directional reflectivity seem to resemble initial changes in retinal ultrastructure (e.g. for RNFL reflectivity investigations [24–28] or to distinguish between PR misalignment or loss [46, 47]), this technique might prove to be a helpful tool for early disease diagnosis.

Furthermore, ophthalmology is not the only target area in which multi-directional OCT might provide valuable input. Also in the fields of oncology and neurology an application may prove beneficial. Since cancerous tissue commonly exhibits differing scattering characteristics compared to healthy tissue, probing from multiple orientations simultaneously might assist in terms of tissue discrimination [58]. In neurodegenerative diseases, such as Alzheimer’s disease, conglomerates of extracellular amyloid beta can be found in some of the brains of patients. These deposits are reported to be highly scattering in comparison to the surrounding tissue [59, 60], possibly due to their fibrous structure or higher density. The analysis of directional scattering in post mortem brain tissue – e.g. using multi-directional OCT – might improve diagnostics of these plaques [61].

## 5. Conclusion

In this work, we demonstrated the concept of multi-directional OCT – a combination of multi-channel and directional OCT – for more detailed investigations of tissues exhibiting angle-dependent optical reflectivity properties. The technique was applied in the ophthalmic environment to study the directional reflectivity of retinal layers in the peripapillary and the macular region. Due to the simultaneous recording of three reflectivity scans from three differently angled orientations, we were able to visualize and contrast directionally reflective retinal tissues – such as the RNFL, HFL as well as parts of the PR layer – within one acquisition. Although this study was restricted to imaging of healthy volunteers, multi-directional OCT might additionally prove its clinical applicability for early diagnosis of ophthalmic diseases.
